# Supplementary material for: Hypothesis: a Plastically Produced Phenotype Predicts Host Specialization and Can Precede Subsequent Mutations in Bacteriophage
Source: mBio. 2018 Nov 13;9(6):e00765-18. doi: 10.1128/mBio.00765-18 (PMC6234872; doi:10.1128/mBio.00765-18)
Supplement: TEXT S1 [file mbo006184163s1.pdf]

Supplementary Information

Hypothesis: A plastically-produced phenotype predicts host-range specialization and can precede subsequent mutations bacteriophage

Colin S. Maxwell

Department of Biology, University of North Carolina, Chapel Hill, NC 27599, USA.

Direct correspondence to: maxwell3@email.unc.edu

Index

Supplementary Methods
Calculating epistasis between methylation and adsorption rate
Detailed description of model
Supplementary Results
The fitness effects of methylation and adsorption rate are epistatic
Calculating event order
Supplementary Figures
Figure S1. DNA methylation and tail fiber affinity are epistatic in the presence of restriction endonucleases.
Figure S2. The predictive power of methylation is robust to a trade-off between p_A and p_B, simulation length, and mutation rate
Figure S3. The predictive power of methylation is robust to imperfect methylation
Figure S4. Methylation can precede mutation for realistic values of restriction bypass and mutation
Supplementary Tables
Table S1. List of parameter values
References

Supplementary methods

Measuring epistasis between methylation and adsorption

Phages can evolve to exploit new hosts by evolving a generalist phenotype (1). However, intuitively, the presence of the distinct R-M systems would seem to preclude the evolution of a generalist. To understand the selective pressures that may cause specialists to evolve, I examined the fitness consequences of gaining traits that help a phage to infect the new bacterial host. I calculated the expected number of offspring from a phage with either the methylation pattern of the new host, greater affinity for the receptors on the new host, or both. I then calculated the selection coefficient for that phage in the ancestral population over a short period of time.

I considered a case in which a species of bacteriophages has strong affinity for receptors on one species of bacteria (bacteria A), and weak affinity for receptors on another (bacteria B). The adsorption rate of a phage for a species of bacteria i (k_i) has units of $(cells \cdot phage \cdot hours \cdot mL)^{-1}$. For these calculations I used a rate of $3 \cdot 10^{-8}$, which is the adsorption rate of T7 phage (2). For these calculations, I assumed an equal mixture of the two bacterial species, which had different receptors and different R-M systems, but were otherwise identical. I assumed that there were many more bacteria than phage, so that the rate of co-infection is negligible.

I calculated fitness the fitness of a single bacteriophage over a convenient unit of time: one hour. The probability of a phage adsorbing to bacterial species i in one hour when i is the only bacterial species is:

$$a_i = P(adsorb\ i) = e^{-kn_i}$$

where n_i is the number of bacteria i in one mL. If two bacterial species are mixed together, then the probability that the phage adsorbs to bacterial species B and not A is given by:

$$p_B = P(adsorb\ B, not\ A) = \frac{a_B \cdot n_B}{a_A \cdot n_A + a_B \cdot n_B}$$

If a phage is marked with a methylation pattern produced by the RM system in bacteria B, the expected number of offspring from bacteria B is equal to the burst size b by probability of infecting B $b \cdot p_B$. The expected number of offspring from bacteria A is $b \cdot (1 - p_B) \cdot r$, where r is the probability of being modified by the methyltransferase before being cleaved by the restriction endonuclease, since otherwise the phage is killed by the individual in bacterial species A that it infects. Adding these together, the expected number of offspring for a bacteriophage marked by methylation pattern B is given by:

$$w = b \cdot (p_B + (1 - p_B) \cdot r\ )$$

I then calculated the selection coefficient for a phage with a particular combination of methylation pattern and tail fiber affinities using $s = 1 - w_1/w_0$, where w_1 is the reproductive output of the phage under consideration and w_0 is the reproductive output of the phage I compared it to.

These calculations indicated that in the presence of two hosts with two R-M systems (1) that methylation patterns and mutations affecting the affinity of the phage for a bacteria are epistatic and (2) that phage are most fit when they bind to a single host.

Detailed description of individual based model

I modeled the evolution of a population of bacteriophage evolving in a well-mixed environment that is constantly fed by a mixture of two species of bacteria (phages and bacteria are generically referred to as 'agents'). I did not model co-evolution between the bacteriophage and the bacteria.

Time. The model was a discrete time model. During each time step the model randomly chose whether all the bacteriophage or all the bacteria were 'activated' first. 'Activated' is jargon from the Mesa framework. Agents are simulated as instances of a class (e.g. Bacteria or Phage) with associated methods. By 'activated' I mean that the 'step' method associated with the instance was called. Within either all instances of Phage or Bacteria (called 'breeds' in the Mesa framework), the agents were activated in a random order. This scheme is called "random activation by breed" in the Mesa framework.

Space. To simulate mixing, at each time step the bacteria and the phages were place randomly on a line [0,1]. Each bacteriophage then encountered all the bacteria within a certain distance of it (the 'encounter width') in a random order until it bound to a bacterium.

Phage traits. I modeled bacteriophages as having two traits: (1) DNA that could be marked with one of two methylation patterns (patterns A and B, named after the bacterial species that creates them), or with no methylation pattern, and (2) tail fibers that would bind to the receptor of a bacterium if it encountered it during a time step with some probability. The probability of binding to a bacterium (p) if it was encountered varied depending on the type of receptor on the bacterium (p_A, p_B) (the 'affinity' for the bacteria).

Steps in infection of bacteria. During each step, bacteriophage encounter bacteria within their encounter width until they bind to one. If the bacteriophage failed to bind to a bacterium within three time steps, the phage was killed. If a phage bound to a bacterium that had already been infected by another phage, then the infecting phage was killed. This is called 'superinfection exclusion,' and is common in bacteriophage species (e.g. (3)). If the phage bound to a bacterium that was not infected, it injected its DNA into the bacteria, which was then possibly degraded by the R-M system of the bacteria (see below). If the DNA was not degraded, the phage parasitized the bacteria whereupon the phage persisted in the bacteria until it entered the lytic cycle. During each step of the model, the phage entered into the lytic cycle with some probability (the 'latency'). If it entered into the lytic cycle, the bacteria was killed and some number of progeny phage (the 'burst size') were produced.

Phage tail fiber mutation. Phage progeny genetically inherited their tail fiber affinity from their parent. When phage progeny were produced, the affinity of their tail fibers for each of the two bacterial species could mutate independently with some probability (the 'mutation frequency'). If they mutated, a fixed number (the 'mutation step size') was randomly either added or subtracted from the affinity of the tail fiber for each of the two bacterial species. Unless otherwise specified, I used a mutation frequency of 10% and a mutation step size of 0.1 because these allowed the

phage to evolve to specialize on their hosts within a manageable amount of simulated time. I verified that the major conclusions of the model were robust to step size, mutation frequency, and simulation length (see below).

It is possible that the ability of a phage tail fiber to bind to one receptor could compromise the ability to bind to a different receptor (e.g. (4)) alternatively, there may not be such a trade-off. If there is a trade-off between the ability to bind to one receptor and another, the shape of this trade-off can affect the outcome of evolution. A convex trade-off, where an increase in the affinity for one receptor is accompanied by a larger decrease in the affinity for another receptor, is expected to select for phage to specialize to infect one species of bacteria (5). To model this, I either allowed the tail fiber affinities to vary independently of each other ('no trade-off', Figure 1), or constrained them using the parametric equations:

$$p_{B_{max}} = (1 - x)^s$$
$$p_{A_{max}} = (x)^s$$
$$x \in [0,1],$$
$$s \in \{1,2\}$$

When $s = 1$, these equations reduce to a straight line with an p_B and p_A intercept of 1 ('flat trade-off', Figure 1). When $s = 2$, these equations create a convex trade-off between p_B and p_A ('convex trade-off', Figure 1). The tail fibers were constrained by moving the affinities of the tail fibers to the closest point on the constraining line if the affinities fell above the line. Note that all main text figures were generated with no trade-off. Only Figure S2 was generated using a trade-off.

Methylation schemes. I modeled the production of methylation patterns on phage genomes according to five schemes. Methylation could be strictly 'plastic.' If methylation is plastic, then progeny are marked with the bacteria's methylation pattern with some probability (the 'methylation efficiency'), otherwise, the progeny are not marked by any methylation pattern. Note that this is the real pattern in nature: the methylation efficiency of phage in the lab varies between ~100% for phage lambda and P2 (6, 7) and 15-50% for T7 (8). I modeled plastic methylation with (1) 100%, (2) 50%, and (3) 10% efficiency. Note that in the latter two cases 50% or 90%, respectively, of phage offspring will have no methylation pattern and will thus be susceptible to restriction from either bacterial species A or B. (4) Methylation could be inherited 'genetically.' In this case, phage progeny would inherit their parent's methylation state as if it were encoded by DNA. In this case, the methylation pattern of a phage mutated with frequency $1 - restriction\ efficiency$ so that it had a similar rate of change as in the plastic case. (3) Methylation could be 'random.' In this case, phage progeny would be randomly assigned methylation pattern A or B with a 50:50 probability. Note that the latter two schemes do not occur in nature, but are included as controls to separate the effects of the MTases from the activities of the REases.

Bacteria traits. I modeled two species of bacteria that differed by the receptors recognized by the bacteriophages and their R-M systems. The receptors of the bacteria were recognized with different affinities by the phage tail fibers. The restriction-modification systems destroyed DNA that was injected by a phage that is not marked with the cognate methylation pattern with some

probability (the 'restriction efficiency'). If the phage was not destroyed by the R-M system, then it was able to parasitize the bacteria.

Supplementary Results

The fitness effects of methylation and adsorption rate are epistatic

Consider a population of bacteriophage that has exclusively infected one of two available species of bacteria which have distinct receptors and restriction-modification (R-M) systems. In this population, the phages have only infected bacteria A, and if we assume that the methylation efficiency of the bacteria is 100%, therefore the DNA of each individual is marked with methylation pattern A. In this scenario the phages adsorb to bacteria A with $k_A = 3 \cdot 10^{-8}$ and to bacteria B with $k_B = 0.05 \cdot k_A$ so that bacteria A is the preferred host due to the greater ability of the phages to bind to it. These parameters are the wild-type adsorption rate of T7 phage to a bacteria and a plausible rate of binding to a different strain (2). If this population of phage were to evolve a population that specialized for bacteria B, they would become methylated with methylation pattern B and would evolve to bind to the receptor on bacteria B with greater affinity. I considered the fitness consequences of each of these events (1) gaining the new methylation pattern, and (2) gaining greater affinity for bacteria B.

I first examined the fitness of a bacteriophage with the same adsorption rate to each bacterial species but with a new methylation pattern B in this population (Figure S1A). In the absence of an R-M system, there is no selective advantage or disadvantage to switching methylation pattern. However, when an R-M system is present, the new methylation pattern is only selected for at high concentrations of bacteria B. This is an intuitive result because at high concentrations of bacteria B, the phage marked with methylation pattern B is more likely to infect B and therefore be able to efficiently produce progeny.

I examined selection for a phage with: (1) $k_B = 0.1 \cdot k_A$, (2) methylation pattern B, (3) $k_B = 0.1 \cdot k_A$ *and* methylation pattern B relative to the rest of the population (Figure S1B). Let p'_A be the probability of binding to A in condition (1), and w' be the expected number of offspring produced by the phage in condition (1). Increasing affinity for B decreases the probability of binding to A, which implies that $p'_A < p_A$. Subtracting the expected number of offspring and rearranging the terms gives:

$$w' - w = (p'_A - r(1 - p'_A)) - \big((1 - p_A) - r \cdot p_A\big)$$
$$w' - w = (p'_A - p_A)(1 - r)$$

since $(p'_A - p_A)$ is always less than 1, and $(1 - r)$ is always greater than 1, increasing affinity for bacteria B is always detrimental when marked with methylation pattern A. However, if the phage has a higher affinity for B in addition to having methylation pattern B, it is always more fit than if phage with only methylation pattern B. This demonstrates that a mutation for increased adsorption to a new bacteria and being marked with the methylation pattern of the R-M system contained by that bacteria are epistatic: the fitness of the phage with both a higher adsorption rate and the new methylation pattern is greater than the sum (or product) of the fitness of phage with either trait alone. This also makes intuitive sense, since injecting DNA into a bacteria containing a R-M system is only likely to be successful if the DNA is already marked with a cognate methylation pattern.

Finally, I examined expected reproductive output for phage marked with either methylation pattern A or methylation pattern B for various combinations of k_A, k_B, and different ratios of bacteria A and B (Figure S1C). For each ratio of bacteria A and B, phage marked with methylation pattern B are expected to produce more offspring than those marked with A at high k_B and k_A. The converse is also true: phage marked with A that have a high k_A and a low k_B are expected to produce more offspring than those marked with methylation pattern B. The set of adsorption rates at which methylation pattern A is favored is larger when bacteria A is denser and vice versa.

Methylation can precede mutation for realistic parameter values

Individual based modeling is generally a heuristic approach that doesn't seek to assign realistic biophysical parameters. Therefore, I used a simple analytic model to model when methylation was more likely to occur before mutation. I examined whether a population founded by a single phage that had historically infected host A was likely to either (1) bypass the R-M system of host B, or (2) to gain a mutation in its tail fiber at a single nucleotide. This assumes that there are a small number of mutations that affect the binding of a phage tail fiber to the host receptor, similar to what has been observed in studies of phage host range (2, 4). The single base pair mutation rate for bacteriophage with dsDNA genomes is ~$7.7 \cdot 10^{-7}$ (9). However, phage tail fibers often mutate at a higher rate than other genes in the phage genome. This elevated mutation rate can be due to a variety of mechanisms such as recombinases that swap genes encoding tail fibers, tandem repeats found within tail fibers, and, dedicated retroelements that mutate specific residues at a much higher rate. In the latter case, the mutation rate across the tail fiber gene is as high as 10^{-3} (10). Rates of RM evasion also vary over a wide range: between 10^{-2} to $\sim 10^{-8}$ (11). To span the range of biological possibility, I examined how the order of events (1) and (2) would vary depending on parameters spanning the ranges above.

These numbers are too small to efficiently model using my individual-based model, therefore, to estimate which event was likely to precede the other, I calculated the probability that a population of phage that started from a single founder with methylation pattern A would either (1) bypass the RM system of methylation pattern B, or (2) get a mutation in the tail fiber of one individual. I sought to understand what factors would influence the probability of either event as the population expanded. I assumed non-overlapping generations. If no phage had successfully infected bacteria B, then the number of phage at generation i (n_i) is:

$$n_i = (p_A \cdot b)^i$$

since all the phage that bound to bacteria B were killed by the RM system in bacteria B. If the probability of getting a mutation is μ, then probability of no phage mutating during this generation is:

$$P(no\ mutation) = (1 - \mu)^{(n_i - n_{i-1})}$$

since $n_i - n_{i-1}$ gives the number of phage produced during this generation. The probability of no phage bypassing the RM during this generation is:

$$P(no\ bypass) = (1 - r)^{(n_i \cdot p_B)}$$

since $n_i \cdot p_B$ gives the expected number of phage to bind to bacteria B during this generation.

Methylation can precede mutation for realistic parameter values of mutation and restriction bypass (Figure S4). If the probability of a single phage getting a mutation that changed its tail fiber affinity is similar to that of a single base-pair mutation in dsDNA phage ($\sim 8 \cdot 10^{-8}$), and if the rate of restriction bypass was on the order of 0.01 (which is similar to the rate of phage lambda bypassing the EcoAI R-M system (11)), then R-M bypass is likely to happen first. For example, by the third generation of phage growth, the probability of bypassing the RM system is ~100% if the adsorption rate to B is 10% of the adsorption rate to A and ~25% if the adsorption rate is 0.1% of the adsorption to A. In contrast, there is only a ~1% probability of any phage having a mutation at this point. However, if the rate of restriction bypass is more similar to the rate at which lambda can bypass EcoKI ($\sim 1 \cdot 10^{-8}$) (11), then mutation is likely to occur in the population first. Note that these calculations don't take into account the odds that the phage with the mutation is then able to bypass the R-M system. Thus, they overestimate the probability of mutation preceding methylation.

References

1. **Bono LM**, **Gensel CL**, **Pfennig DW**, **Burch CL**. 2012. Competition and the origins of novelty: experimental evolution of niche-width expansion in a virus. Biol Lett **9**:rsbl20120616–20120616.

2. **Heineman RH**, **Springman R**, **Bull JJ**. 2008. Optimal foraging by bacteriophages through host avoidance. The American Naturalist **171**:E149–57.

3. **McAllister WT**, **Barrett CL**. 1977. Superinfection exclusion by bacteriophage T7. J Virol **24**:709–711.

4. **Meyer JR**, **Dobias DT**, **Medina SJ**, **Servilio L**, **Gupta A**, **Lenski RE**. 2016. Ecological speciation of bacteriophage lambda in allopatry and sympatry. Science **354**:1301–1304.

5. **Levins RT**. 1962. Theory of Fitness in a Heterogeneous Environment. I. The Fitness Set and Adaptive Function. The American Naturalist **96**:361–373.

6. **Bertani G**, **Weigle JJ**. 1953. Host controlled variation in bacterial viruses. J Bacteriol **65**:113–121.

7. **Pleška M**, **Qian L**, **Okura R**, **Bergmiller T**, **Wakamoto Y**, **Kussell E**, **Guet CC**. 2016. Bacterial Autoimmunity Due to a Restriction-Modification System. Curr Biol **26**:404–409.

8. **Studier FW**. 1975. Gene 0.3 of bacteriophage T7 acts to overcome the DNA restriction system of the host. Journal of Molecular Biology **94**:283–295.

9. **Drake JW**, **Charlesworth B**, **Charlesworth D**, **Crow JF**. 1998. Rates of Spontaneous Mutation. Genetics **148**:1667–1686.

10. **Medhekar B**, **Miller JF**. 2007. Diversity-generating retroelements. Curr Opin Microbiol **10**:388–395.

11. **Pleška M**, **Lang M**, **Refardt D**, **Levin BR**, **Guet CC**. 2018. Phage–host population dynamics promotes prophage acquisition in bacteria with innate immunity. Nat Ecol Evol **2**:359–366.
