# Supplementary figures and images for: Hypothesis: a Plastically Produced Phenotype Predicts Host Specialization and Can Precede Subsequent Mutations in Bacteriophage
Source: mBio. 2018 Nov 13;9(6):e00765-18. doi: 10.1128/mBio.00765-18 (PMC6234872; doi:10.1128/mBio.00765-18)

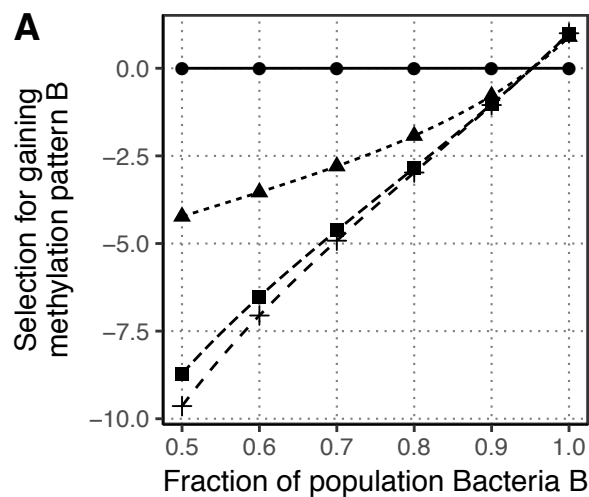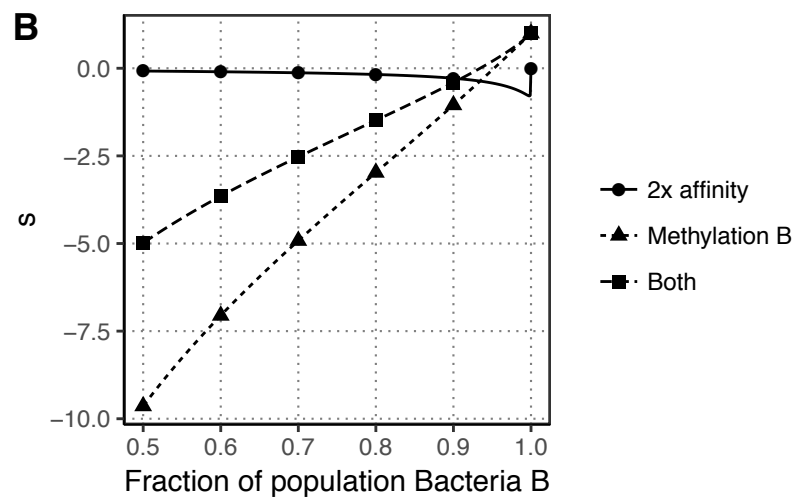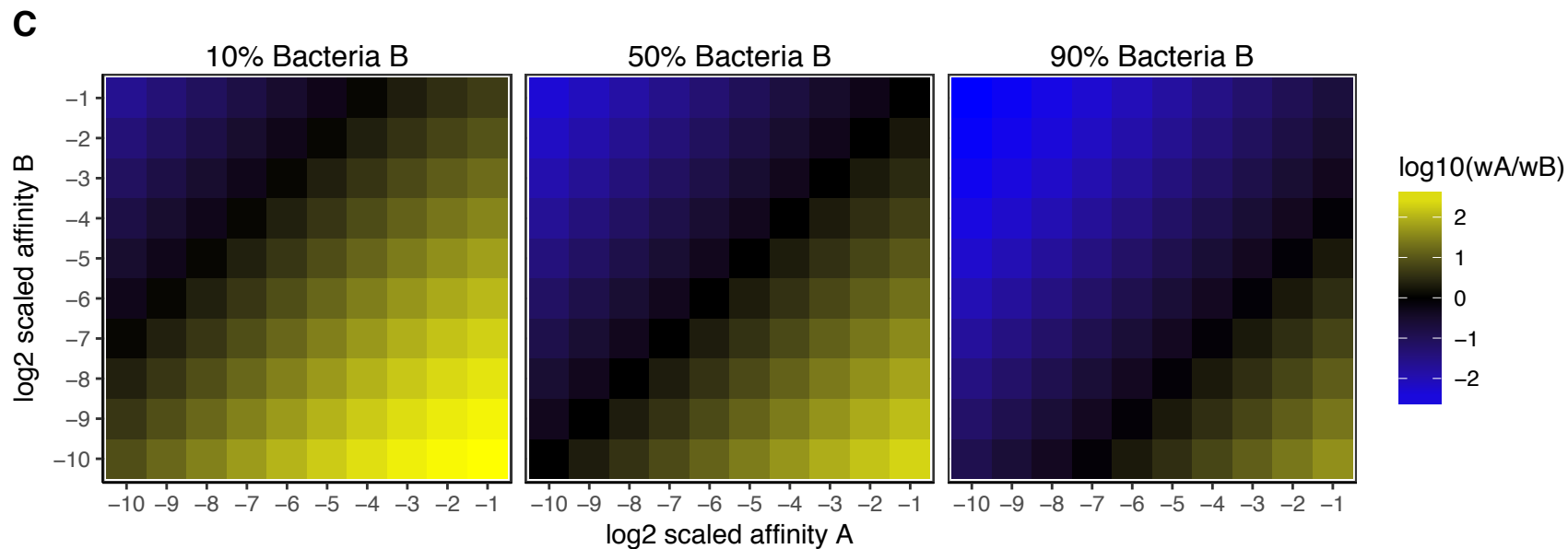

Supplement: FIG S1 [file mbo006184163sf1.pdf]

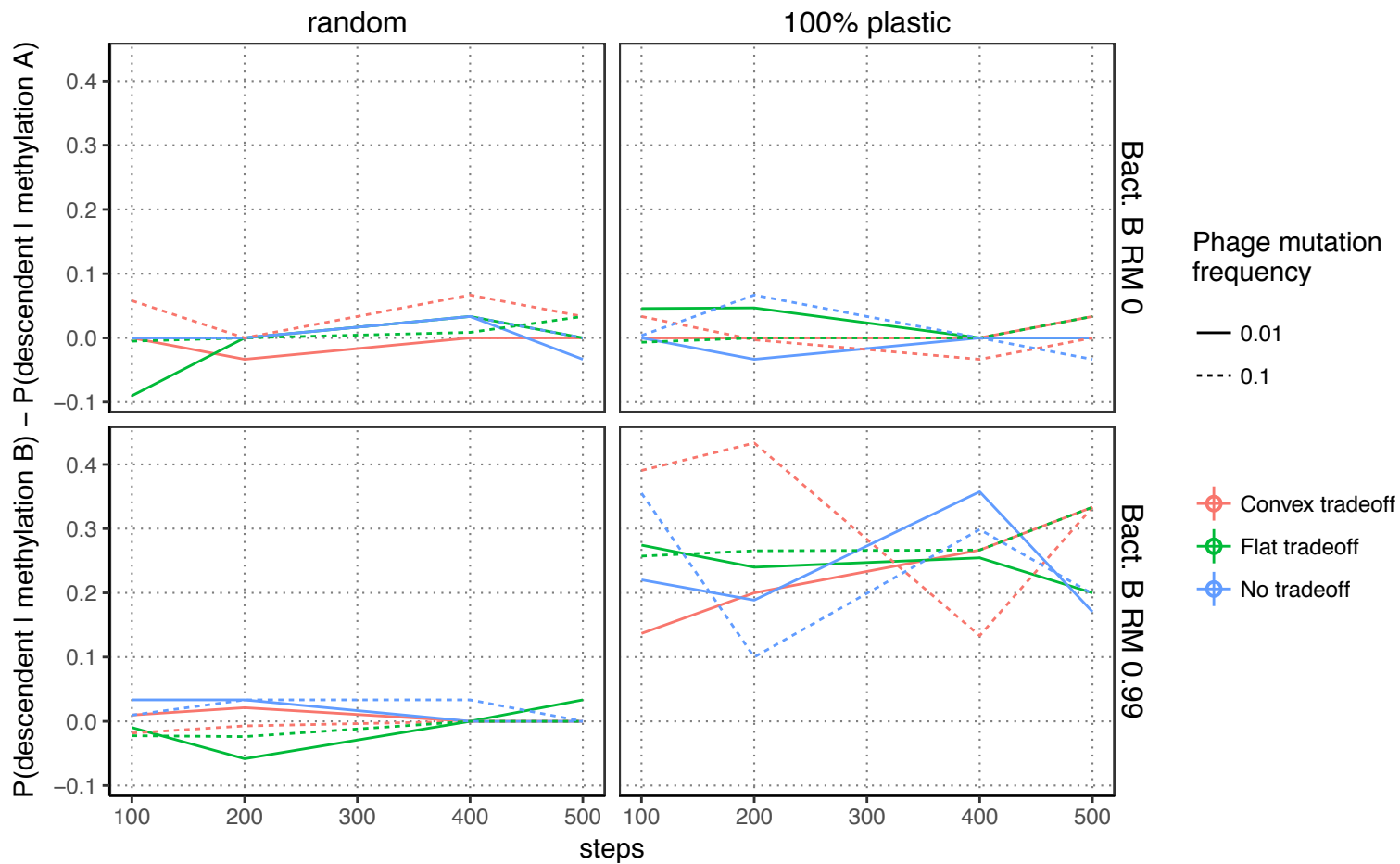

Supplement: FIG S2 [file mbo006184163sf2.pdf]

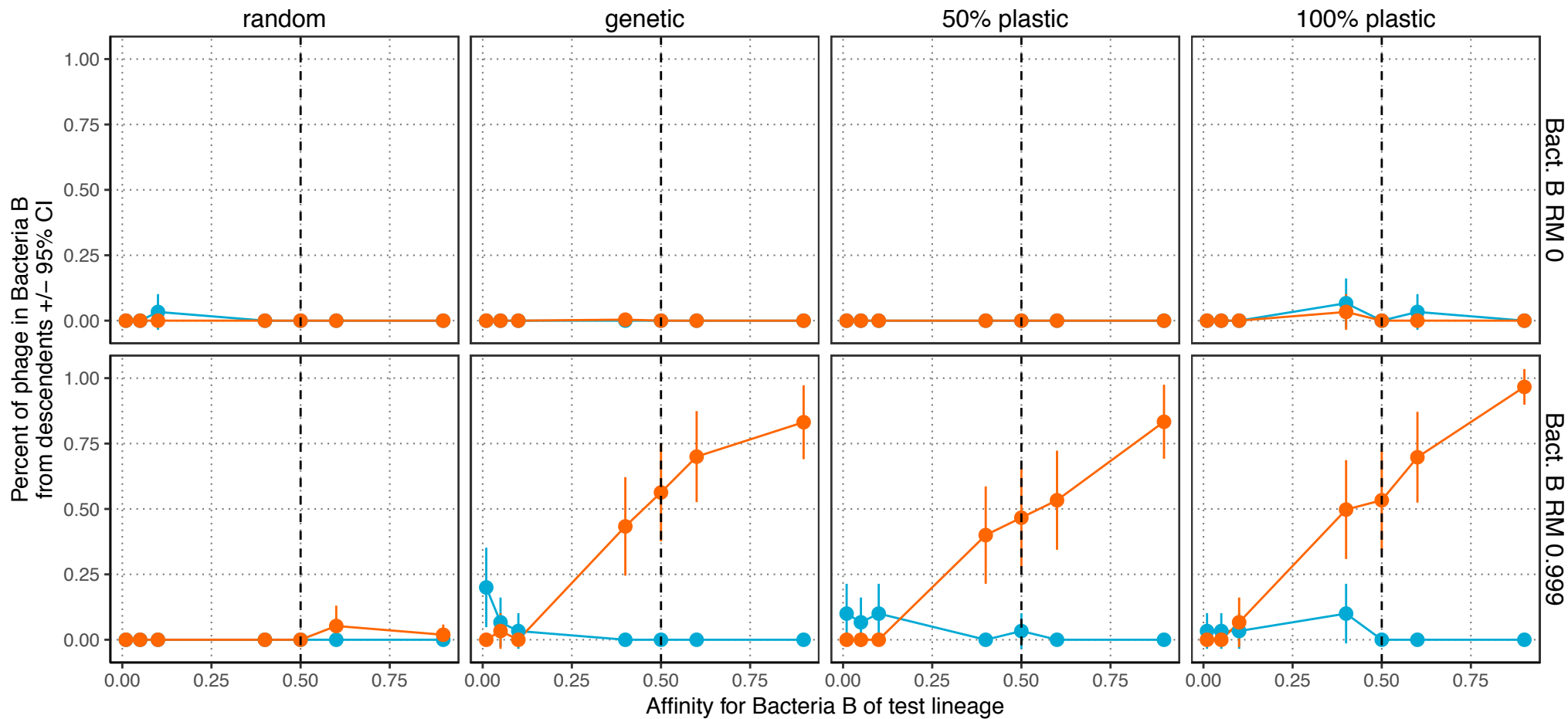

Supplement: FIG S3 [file mbo006184163sf3.pdf]

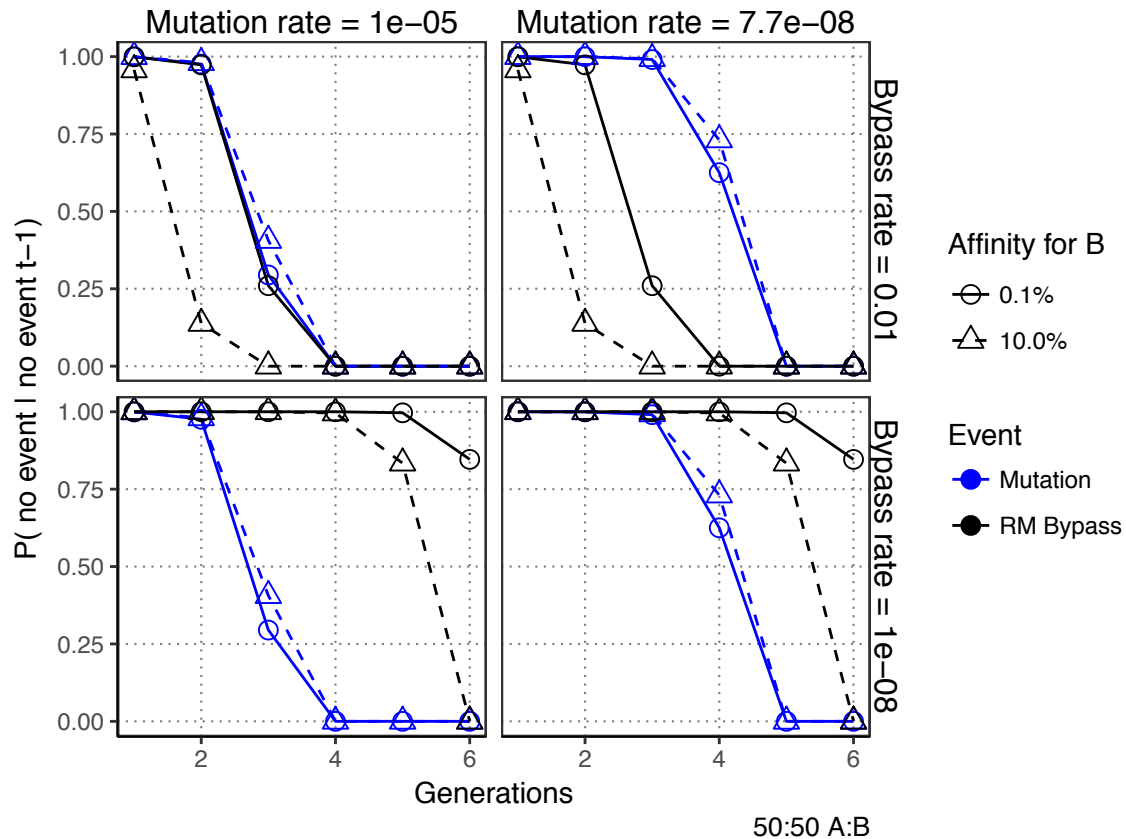

Supplement: FIG S4 [file mbo006184163sf4.pdf]
