# Supplementary material for: Hypothesis: a Plastically Produced Phenotype Predicts Host Specialization and Can Precede Subsequent Mutations in Bacteriophage
Source: mBio. 2018 Nov 13;9(6):e00765-18. doi: 10.1128/mBio.00765-18 (PMC6234872; doi:10.1128/mBio.00765-18)
Supplement: TABLE S1 [file mbo006184163st1.pdf]

Supplementary Table 1. Parameters used in the model.

Parameter name	*Parameter description*	*Values taken*
bacteria_per_step	Number of bacteria added per step	10
encounter_width	Distance that phage encounter bacteria	0.01
fraction_b_m1	Fraction of bacteria with methylation pattern 1 added per step	0.5
initial_bacteria	Number of bacteria at start	100
initial_fraction_p_g1	Fraction of phage with 'genotype' 1. Note that the phage genotype is never different than the methylation pattern, so this was never really used.	1
initial_fraction_p_m1	Fraction of phage with methylation 1	1
initial_phage	Number of phage at start	1000
latency	The odds of entering the lytic cycle during one step if the phage has infected a bacteria.	0.5
phage_burst_size	The number of progeny produced by the phage during the lytic cycle	10
phage_inactivation_time	The number of steps a free phage will persist for unless it binds to a bacteria	3
phage_mutation_freq	The odds that a phage will have a mutation affecting the affinity of the phage for bacteria A or B when it is being produced	0.01, 0.1
phage_mutation_step	The size of the mutations affecting the pA and pB	0.1
phage_off_diagonal	The beginning affinity of phage for the type of bacteria that they do not have the methylation pattern for.	0.05, 0.5
re_degrade_foreign_0	The odds of bacteria 0 degrading improperly methylated DNA	0, 0.99, 0.999
re_degrade_foreign_1	The odds of bacteria 1 degrading improperly methylated DNA	0, 0.99, 0.999
inheritance	The way that phage get methylated.	"genetic", "random", "plastic 100",

		"plastic 50", "plastic 10"
spike_in_affinity_0	The affinity for bacteria 0 of the test lineage	0.1, 0.4, 0.5, 0.6, 0.9
spike_in_methylation	The methylation status of the test lineage	0,1
shape	The trade-off between p_A and p_B	0,1,2
steps	Number of steps simulation runs for	100, 200, 400, 500
